# Independent real‐world application of a clinical‐grade automated prostate cancer detection system

**DOI:** 10.1002/path.5662

**Published:** 2021-04-27

**Authors:** Leonard M da Silva, Emilio M Pereira, Paulo GO Salles, Ran Godrich, Rodrigo Ceballos, Jeremy D Kunz, Adam Casson, Julian Viret, Sarat Chandarlapaty, Carlos Gil Ferreira, Bruno Ferrari, Brandon Rothrock, Patricia Raciti, Victor Reuter, Belma Dogdas, George DeMuth, Jillian Sue, Christopher Kanan, Leo Grady, Thomas J Fuchs, Jorge S Reis‐Filho

**Affiliations:** ^1^ Grupo Oncoclinicas Sao Paulo Brazil; ^2^ Instituto Mario Penna Belo Horizonte Brazil; ^3^ Paige New York NY USA; ^4^ Department of Medicine and Human Oncology and Pathogenesis Program Memorial Sloan Kettering Cancer Center New York NY USA; ^5^ Department of Pathology Memorial Sloan Kettering Cancer Center New York NY USA; ^6^ Stat One Wilmington NC USA

**Keywords:** artificial intelligence, histopathology, diagnosis, screening, prostate cancer, diagnosis, deep learning, machine learning

## Abstract

Artificial intelligence (AI)‐based systems applied to histopathology whole‐slide images have the potential to improve patient care through mitigation of challenges posed by diagnostic variability, histopathology caseload, and shortage of pathologists. We sought to define the performance of an AI‐based automated prostate cancer detection system, Paige Prostate, when applied to independent real‐world data. The algorithm was employed to classify slides into two categories: benign (no further review needed) or suspicious (additional histologic and/or immunohistochemical analysis required). We assessed the sensitivity, specificity, positive predictive values (PPVs), and negative predictive values (NPVs) of a local pathologist, two central pathologists, and Paige Prostate in the diagnosis of 600 transrectal ultrasound‐guided prostate needle core biopsy regions (‘part‐specimens’) from 100 consecutive patients, and to ascertain the impact of Paige Prostate on diagnostic accuracy and efficiency. Paige Prostate displayed high sensitivity (0.99; CI 0.96–1.0), NPV (1.0; CI 0.98–1.0), and specificity (0.93; CI 0.90–0.96) at the part‐specimen level. At the patient level, Paige Prostate displayed optimal sensitivity (1.0; CI 0.93–1.0) and NPV (1.0; CI 0.91–1.0) at a specificity of 0.78 (CI 0.64–0.89). The 27 part‐specimens considered by Paige Prostate as suspicious, whose final diagnosis was benign, were found to comprise atrophy (*n* = 14), atrophy and apical prostate tissue (*n* = 1), apical/benign prostate tissue (*n* = 9), adenosis (*n* = 2), and post‐atrophic hyperplasia (*n* = 1). Paige Prostate resulted in the identification of four additional patients whose diagnoses were upgraded from benign/suspicious to malignant. Additionally, this AI‐based test provided an estimated 65.5% reduction of the diagnostic time for the material analyzed. Given its optimal sensitivity and NPV, Paige Prostate has the potential to be employed for the automated identification of patients whose histologic slides could forgo full histopathologic review. In addition to providing incremental improvements in diagnostic accuracy and efficiency, this AI‐based system identified patients whose prostate cancers were not initially diagnosed by three experienced histopathologists. © 2021 The Authors. *The Journal of Pathology* published by John Wiley & Sons, Ltd. on behalf of The Pathological Society of Great Britain and Ireland.

## Introduction

Diagnostic surgical pathology remains the ‘gold standard’ for cancer diagnosis, despite the challenges posed by the shortage of diagnostic pathologists [1], the difficulty in detecting small quantities of cancer in biopsy material, and non‐trivial levels of inter‐observer variability [2, 3]. These problems may be mitigated by applying artificial intelligence (AI)‐based systems on digital whole‐slide images (WSIs) of histopathology sections to provide greater diagnostic accuracy [4, 5, 6, 7, 8, 9], reduce inter‐observer variability, and alleviate the pressure on pathologists who face increasing caseloads and a decreasing qualified workforce [4, 5, 6, 7, 8, 9].

One of the potential applications of AI is to increase the accuracy and the efficiency of histopathologic assessment of transrectal ultrasound‐guided (TRUS) prostate biopsies, where the false‐negative diagnosis rate for prostate needle biopsies is 1–4% [3, 10, 11]. To address this unmet need, AI‐based algorithms to detect prostate cancer in diagnostic biopsies have been developed [7, 12, 13, 14]. Paige Prostate was developed based on the system described in Campanella *et al* [7]. This clinical‐grade deep learning AI test has recently been granted Breakthrough Designation by the United States Food and Drug Administration (FDA) for the automated detection of cancer in prostate biopsies.

To define the impact of this AI‐based test on histopathology practice, we applied Paige Prostate to real‐world data from a diagnostic histopathology laboratory located in a different country, not involved in the original development and validation of the system. The aims of this study were to assess the diagnostic performance of this AI system in WSIs of TRUS prostate biopsies, to define its impact on the accuracy of board‐certified pathologists interpreting these WSIs, and to evaluate its impact on the diagnostic accuracy and efficiency of experienced diagnostic pathologists. We posited that Paige Prostate would successfully identify the slides where no malignancy was present, thereby optimizing the time pathologists allocate to the analysis of slides containing tumor or posing diagnostic challenges.

## Materials and methods

### Patients and histopathology analysis

A total of 600 previously diagnosed unique TRUS prostate needle core biopsy regions (henceforth referred to as ‘part‐specimens’) from 100 consecutive patients accessioned between 9 May 2019 and 22 August 2019 were retrieved from the pathology archives of the Instituto Mario Penna in Brazil. The clinical details of the patients were not retrieved prior to anonymization. Table 1 summarizes the available clinical and pathological characteristics of the patients. The local ethics committee of the Instituto Mario Penna approved this study, and patient consents were obtained according to the approved protocol. Each patient underwent TRUS prostate needle core biopsies from six prostate regions (for each region, the biopsy procedure aimed to needle target the tissue twice, resulting in approximately 12 cores per patient), which amounted to 6–9 slides per patient (total of 682 glass slides). The diagnoses for these patients were rendered by the local pathologist (PGOS – board certified in Brazil for 20 years) at the Instituto Mario Penna. The available hematoxylin and eosin (H&E)‐stained histologic sections were retrieved from the hospital archives, verified to meet staining quality standards for optical microscopy reading, and re‐reviewed independently by two independent pathologists (EMP and LMS, hereby referred to as central pathologists) board certified in Brazil for 27 and 16 years, respectively. Their review was also blinded to the original diagnoses. Next, the clinical H&E‐stained sections mounted on glass slides were scanned using a Leica AT2 scanner (Leica Biosystems, Division of Leica Microsystems Inc, Buffalo Grove, IL, USA) at 20× [0.5 μm per pixel (mpp)] and 40× (0.25 mpp) magnifications/resolutions, generating WSIs in the SVS file format. The central pathologists completed WSI review using Aperio Imagescope 64‐bit digital slide viewer v12.4.3 (Leica Biosystems) and a Dell 21‐in. high‐resolution monitor (Dell Inc, Eldorado do Sul – RS, Brasil). The WSIs scanned at 40× (0.25 mpp) magnification/resolution were employed by the central pathologists for the re‐review. The central pathologists independently classified the WSIs generated for each TRUS prostate needle core biopsy region into one of the following categories: benign, malignant or suspicious [i.e. not definitively classifiable without additional action including immunohistochemical (IHC) staining, consensus opinion, second opinion or additional levels]. Discrepancies between diagnoses rendered by each pathologist were adjudicated by subsequent re‐review of the H&E WSIs at 40× such that in addition to the individual pathologist's interpretation, one final diagnostic interpretation agreed upon by both pathologists was rendered for each WSI. For WSIs with compromised viewing quality, the glass slides were reviewed (approximately <5% of all slides).

**Table 1:** Clinicopathologic characteristics of the patients included in the study.

	Patients without cancer	Patients with cancer	All patients
Total patients, *n*	50	50	100
Patient age (years)			
*n*	50	50	100
Mean (SD)	64.9 (7.23)	68.6 (7.65)	66.8 (7.63)
Median	65	69	67
Min–max	48–77	47–84	47–84
Patient age (years) category			
*n*	50	50	100
≤49	1 (2.0%)	1 (2.0%)	2 (2.0%)
50–54	2 (4.0%)	0 (0.0%)	2 (2.0%)
55–59	10 (20.0%)	4 (8.0%)	14 (14.0%)
60–64	10 (20.0%)	11 (22.0%)	21 (21.0%)
65–69	13 (26.0%)	10 (20.0%)	23 (23.0%)
≥70	14 (28.0%)	24 (48.0%)	38 (38.0%)
Patient ISUP GG category			
*n*	50	50	100
Benign	50 (100.0%)	0 (0.0%)	50 (50.0%)
ISUP GG 1 (3 + 3)	0 (0.0%)	7 (14.0%)	7 (7.0%)
ISUP GG 2 (3 + 4)	0 (0.0%)	18 (36.0%)	18 (18.0%)
ISUP GG 3 (4 + 3)	0 (0.0%)	12 (24.0%)	12 (12.0%)
ISUP GG 4 (4 + 4, 3 + 5, 5 + 3)	0 (0.0%)	6 (12.0%)	6 (6.0%)
ISUP GG 5 (4 + 5, 5 + 4, 5 + 5)	0 (0.0%)	7 (14.0%)	7 (7.0%)
Patient PSA (ng/ml)			
*n*	44	43	87
Mean (SD)	8.0 (4.84)	214.9 (924.8)	110.2 (654.6)
Median	7	9	8
Min–max	1–31	3–5635	1–5635
Patient PSA (ng/ml) category			
*n*	44	43	87
<3	3 (6.8%)	0 (0.0%)	3 (3.4%)
3≤5	7 (15.9%)	8 (18.6%)	15 (17.2%)
5≤10	26 (59.1%)	17 (39.5%)	43 (49.4%)
≥10	8 (18.2%)	18 (41.9%)	26 (29.9%)

NA – Gleason grade and tumor size are not applicable to negative patients/slides.

Paige Prostate 1.0 is a convolutional neural network (CNN) based on the multiple instance learning algorithm presented in Campanella *et al* [7]. An early version (‘Paige Prostate Alpha’) was described in Raciti *et al* [15], but the system used and described here is a later version. Paige Prostate 1.0 learns directly from diagnosis without the need for pixel‐wise annotation. Paige Prostate 1.0 runs on WSIs at 20× resolution. For higher resolutions, Paige Prostate 1.0 will use the 20× level of the WSI or it will downsample a higher resolution to 20× if a 20× level is not available (see supplementary material, Supplementary materials and methods).

**Figure 1:**
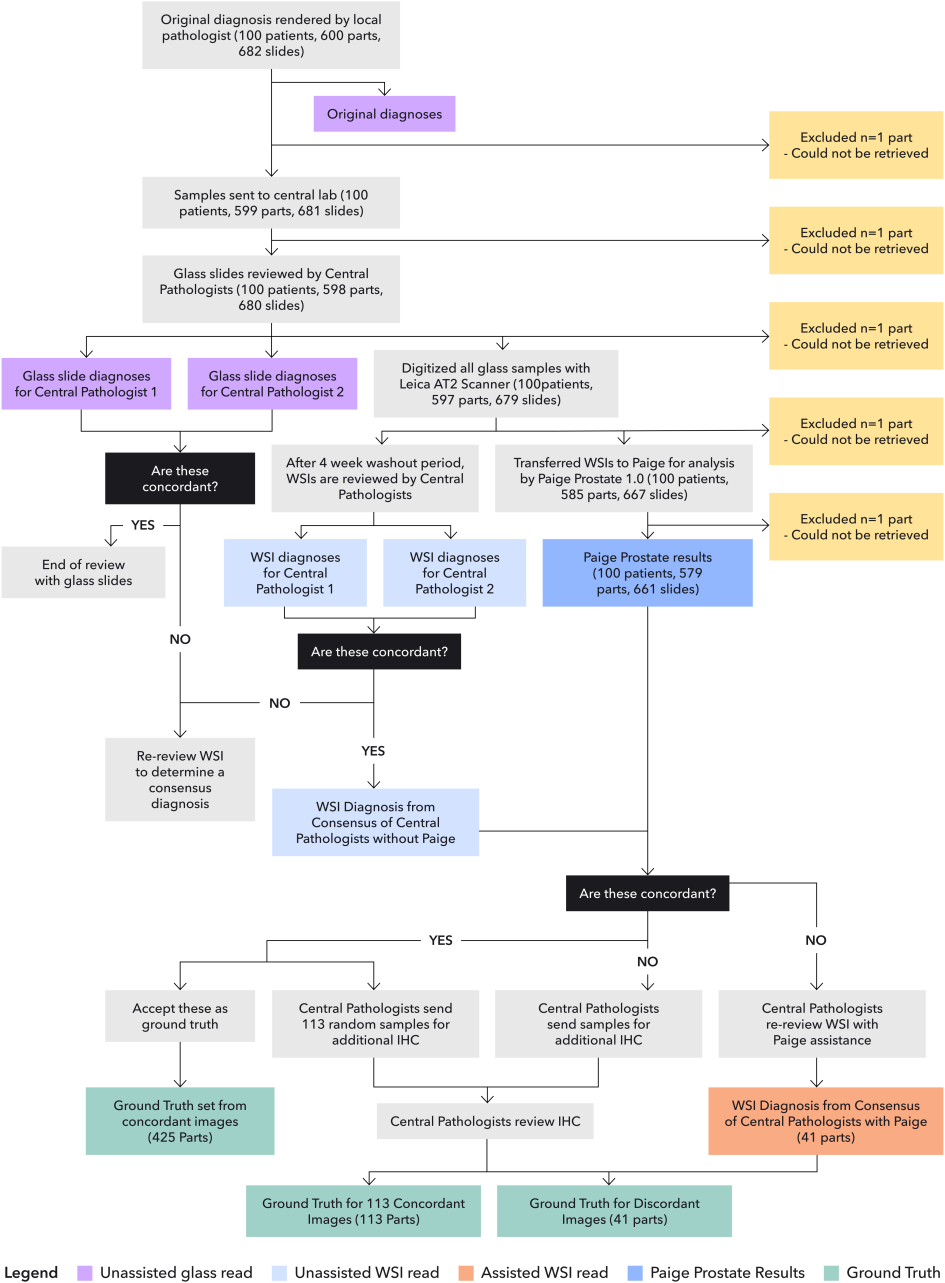
Study flow chart detailing the cases, slides and parts analyzed, and the definition of ground truth utilized in the study.

Paige Prostate generated binary predictions (benign or suspicious for cancer), blinded to clinical and pathologic information. In this classification, benign would prompt no further action by the pathologist, whereas a classification of suspicious would result in further histologic analysis and/or additional IHC to rule out the presence of malignancy. For WSIs predicted as suspicious, Paige Prostate pointed to a region with the highest likelihood for harboring cancer, along with a heatmap visualization. Paige Prostate results were generated for 661 WSIs from 579 unique prostate needle core biopsy parts. From the 682 slides at the original hospital, one could not be retrieved; one slide was broken and could not be scanned; one was not scanned by the scanner; 12 images were not transferred to Paige; and six images were corrupted and could not be analyzed (Figure 1).

Performance analysis was completed at the prostate needle core biopsy ‘part‐specimen level’ to emulate how pathology reports are rendered in practice. If a part‐specimen consisted of more than one glass slide or WSI, the diagnosis of the central pathologists was based on the combined histopathological features seen on all slides or WSIs available for that specific prostate needle core biopsy. Similarly, if Paige Prostate considered any of the available WSIs in a part‐specimen as suspicious, that part‐specimen was classified by Paige Prostate as suspicious, and if all available WSIs in a part‐specimen were benign, that part‐specimen was classified by Paige Prostate as benign. All data were de‐identified prior to conducting the analysis using Paige Prostate.

One hundred concordant part‐specimens (classification by Paige and that of the consensus diagnosis of the central pathologists were concordant) were re‐reviewed by an independent general pathologist (PR). In addition, discordant part‐specimens between Paige Prostate or the consensus of the central pathologists and the final ground truth were digitally re‐reviewed by an expert GU pathologist (VR).

The WSIs included in this study and the consensus diagnoses of two central pathologists are available using Microsoft Teams (Microsoft Inc, Redmond, WA, USA). Those interested in accessing the images can complete the request form https://bit.ly/36cPf6k.

### Statistical analysis

For statistical analysis, a negative was defined as benign and a positive as malignant or suspicious. For a part‐specimen (i.e. specific region targeted by a TRUS prostate needle core biopsy) to be negative, all WSIs in the part‐specimen must be negative.

The following procedure was used to assign the ground truth labels to each part‐specimen. If the consensus of the central pathologists and Paige Prostate agreed, then this classification was assigned as the ground truth for the part‐specimen; otherwise, additional histologic sections of the corresponding part‐specimen were cut and subjected to IHC analysis and reinterpretation by the pathologists to assign the final ground truth (Figure 1). IHC analysis is regularly performed for prostate biopsies when a definitive diagnosis cannot be made from H&E alone [3] and was also performed in 113 randomly selected slides related to 20 patients where the diagnoses were concordant between the local pathologist, central pathologists, and Paige Prostate to ensure the accuracy of the ground truth diagnoses (supplementary material, Supplementary materials and methods). IHC was performed utilizing ready‐to‐use antibodies against high‐molecular‐weight cytokeratin (ready‐to‐use, clone 34βE12; DAKO, Glostrup, Denmark), p63 (ready‐to‐use, clone 4A4; DAKO), and P504S (ready‐to‐use, clone 13H4; DAKO) on an Agilent Autostainer Link 48 system (DAKO) following DAKO PT link (DAKO) antigen retrieval using Tris–EDTA buffer (pH 9.0). A total of 200 μl of ready‐to‐use primary antibody was used per slide. External controls (basal cells on normal prostate and prostate cancer tissues) were used, and internal controls were also checked.

Statistical analysis was performed (by GD) following the pre‐specified definitions agreed upon by the study team, with one of the authors acting as the honest broker (JSR‐F). Paige did not have any direct access to the final integrated data prior to the ‘data freeze’ for the statistical analysis. The analyses were completed (i) by treating each part‐specimen (i.e. specific region targeted by a TRUS prostate needle core biopsy) as independent (i.e. part‐specimen level) and (ii) by treating the diagnosis for a patient based on all parts for a given patient (i.e. patient level). *P* values less than or equal to 0.05 on a two‐sided exact binomial test were considered significant and two‐sided 95% confidence intervals (CIs) were calculated. This study is compliant with the REMARK guidelines (supplementary material, Table S1).

## Results

A ground truth diagnosis, based on the histological analysis by the local pathologist, the two central pathologists, Paige Prostate, and additional histologic review and IHC analysis of all parts where there was a disagreement between diagnosis rendered by Paige Prostate and the consensus of central pathologists, could be rendered for 579 of the original 600 parts (Figure 1). In addition, IHC analysis of the 113 randomly selected slides with concordant diagnoses between the local pathologist, central pathologists, and Paige Prostate confirmed the ground truth diagnoses in all cases (data not shown).

**Figure 2:**
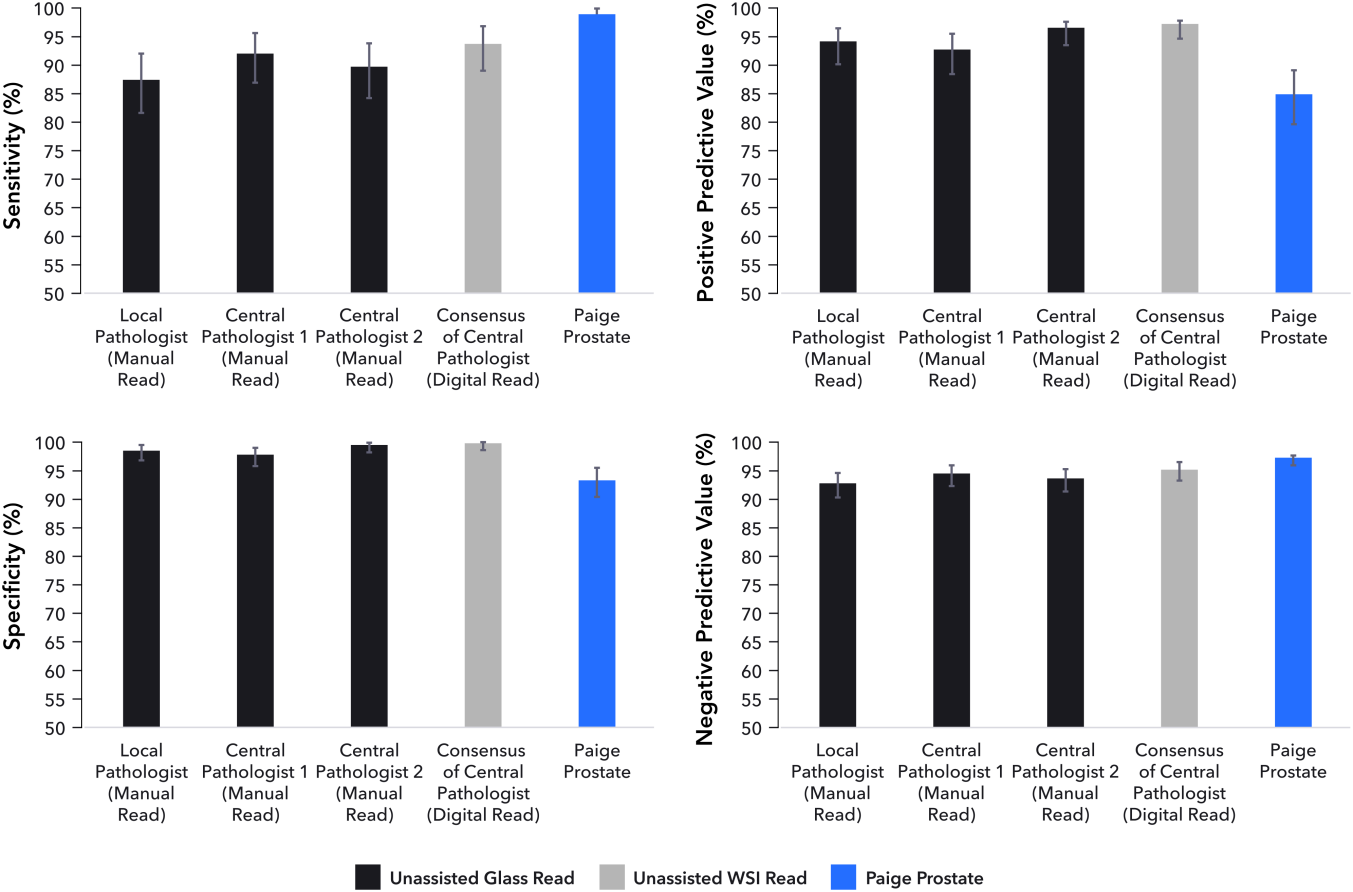
Sensitivity, specificity, positive predictive value (PPV), and negative predictive value (NPV) at the part‐specimen level of the local pathologist, individual central pathologists, the consensus of central pathologists, and Paige Prostate.

Paige Prostate classified 200/579 (34.60%) of the parts as suspicious for cancer and 379/579 (65.46%) as benign. Based on the ground truth diagnoses, compared with the local pathologist, the individual central pathologists, and the consensus diagnoses of the central pathologists (Figure 2 and supplementary material, Table S2), Paige Prostate displayed a favorable sensitivity (0.99; CI 0.96–1.0) and NPV (1.0; CI 0.98–1.0) at the part‐specimen level, while maintaining an acceptable level of specificity (0.93; CI 0.90–0.96). These findings are consistent with our hypothesis that Paige Prostate could accurately identify the parts containing cancer as suspicious (i.e. needing additional review), without flagging a disproportionately high number of parts as suspicious.

**Figure 3:**
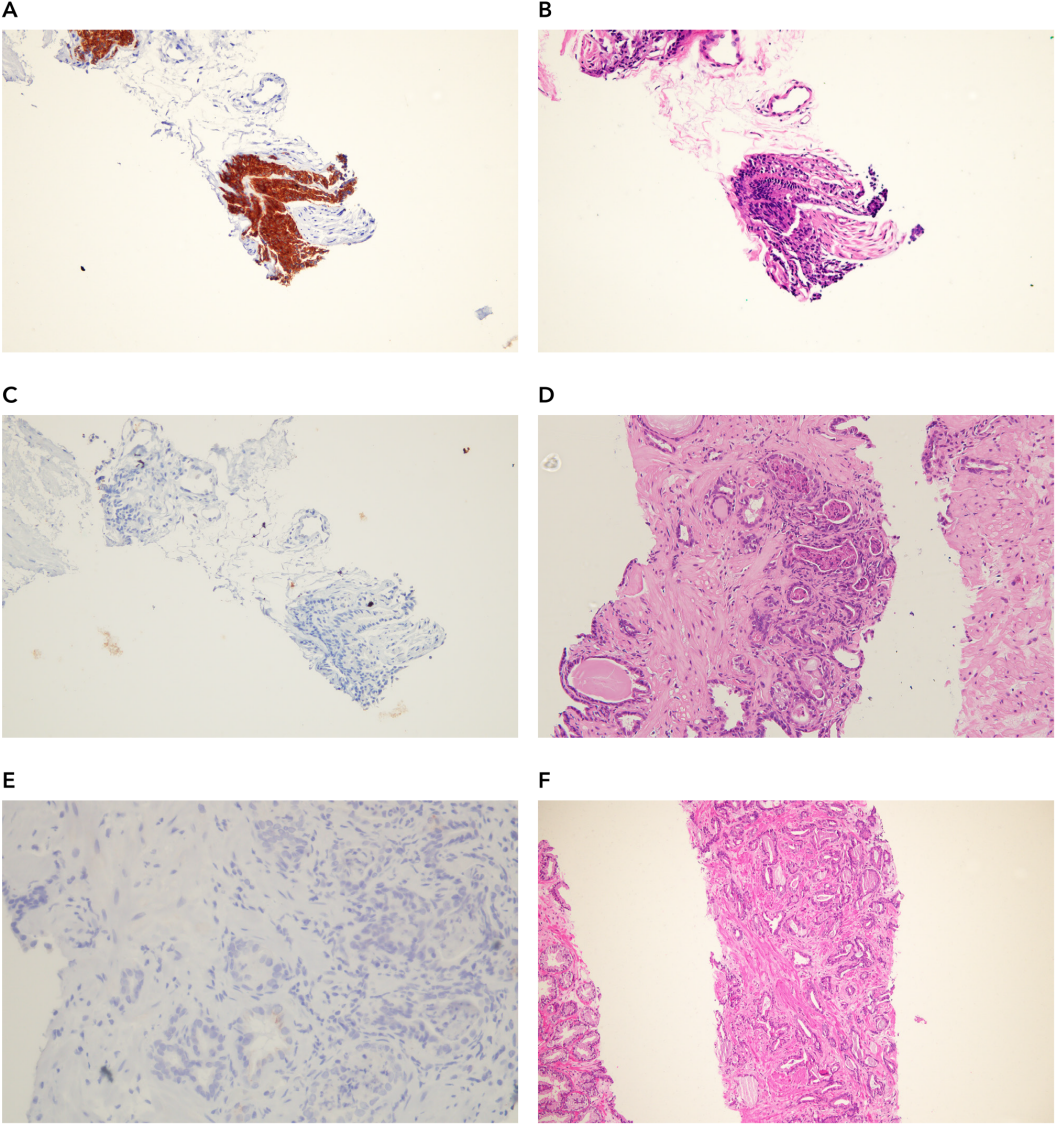
Histologic features of prostate biopsies where Paige Prostate rendered a diagnosis of benign where the ground truth diagnosis was malignant. (A) Representative areas of an ultrasound‐guided transrectal prostate biopsy which Paige Prostate considered benign, yet the ground truth diagnosis was malignant. There is adenocarcinoma with extra‐prostatic extension and perineural invasion (WSI 1002529). (B) Expression of P504S in areas of adenocarcinoma shown in A. (C) Lack of expression of HMWC – 34βE12 in areas of adenocarcinoma shown in A. (D) Representative area of a TRUS prostate biopsy which Paige Prostate considered benign, yet the ground truth diagnosis was malignant (WSI 1002523). (E) Lack of expression of HMWC – 34βE12 in areas of adenocarcinoma shown in D. (F) Invasive prostatic adenocarcinoma in a different biopsy region (i.e. part‐specimen) of the same patient depicted in D (WSI 1002522).

Of the 579 part‐specimens analyzed, 42 discordant results were observed between the consensus diagnosis by the central pathologists and Paige Prostate. For three part‐specimens (3/579, 0.52%), Paige Prostate rendered a diagnosis of benign, whereas the original consensus diagnosis of the central pathologists was ‘suspicious’ or ‘malignant’. Upon re‐review with IHC, two of them were indeed prostate cancer; one contained only extra‐prostatic extension of prostatic adenocarcinoma harboring a focus of perineural invasion (Figure 3A–C) and another had prostatic adenocarcinoma with an atrophic appearance (Figure 3D,E). In the former false negative, the remaining part‐specimens from that patient were correctly found to be suspicious for carcinoma by Paige Prostate. In the second false negative, one other part‐specimen from that patient was correctly identified by Paige Prostate as suspicious (Figure 3F). In the third case where Paige Prostate rendered a diagnosis of benign whereas the original consensus diagnosis of the pathologists was ‘suspicious’, the findings of IHC confirmed that Paige Prostate was correct in classifying it as ‘benign’.

**Figure 4:**
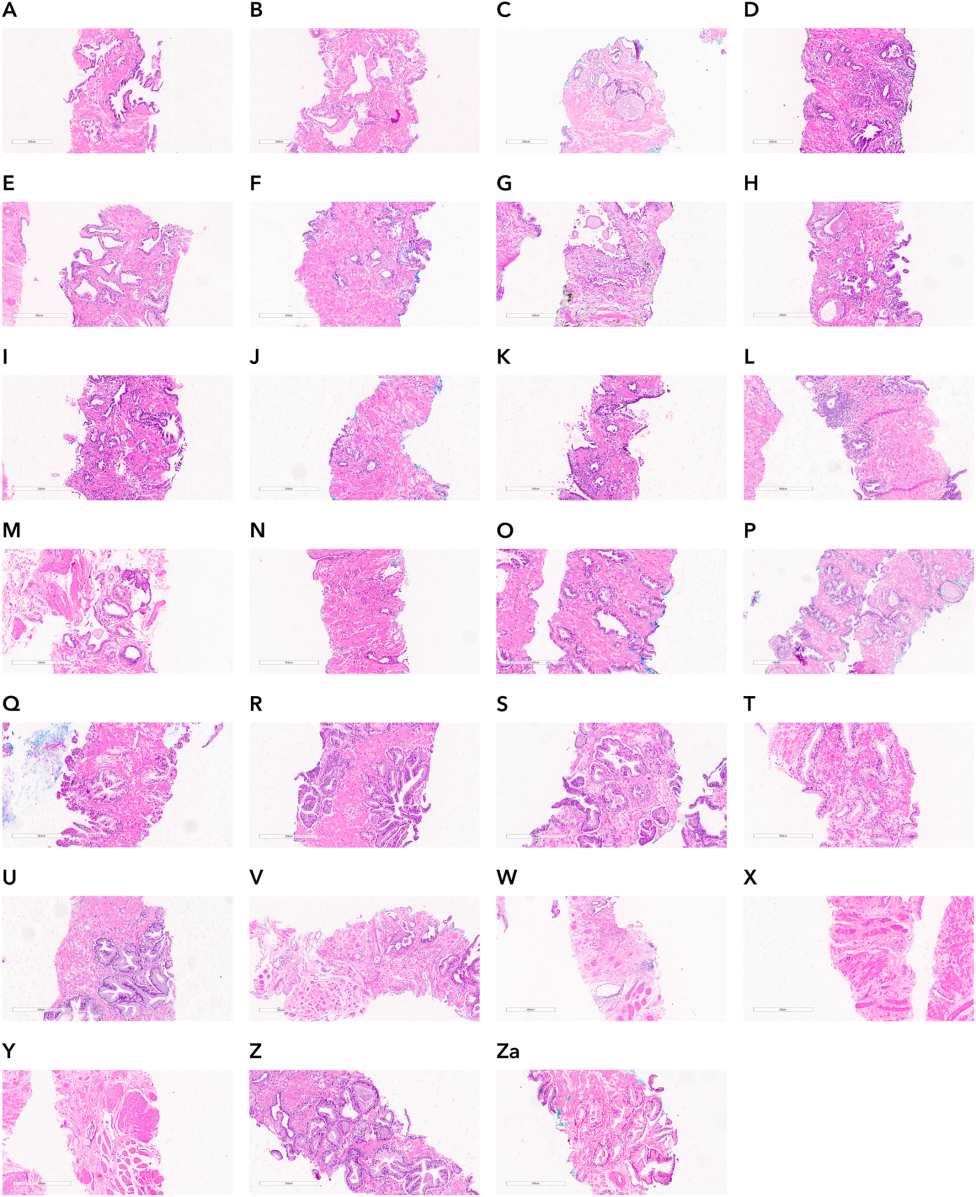
Histologic features of prostate biopsies where Paige Prostate rendered a diagnosis of suspicious when the ground truth was benign. Representative areas of 27 slides classified as suspicious by Paige Prostate and the ground truth was benign. (A–M) Prostate tissue with foci of atrophy. (N) Post‐atrophic hyperplasia. (O–T) Benign prostate tissue. (U) Atrophy and apical benign prostate tissue. (V–X) Apical benign prostate tissue. (Y–Za) Benign adenosis.

**Figure 5:**
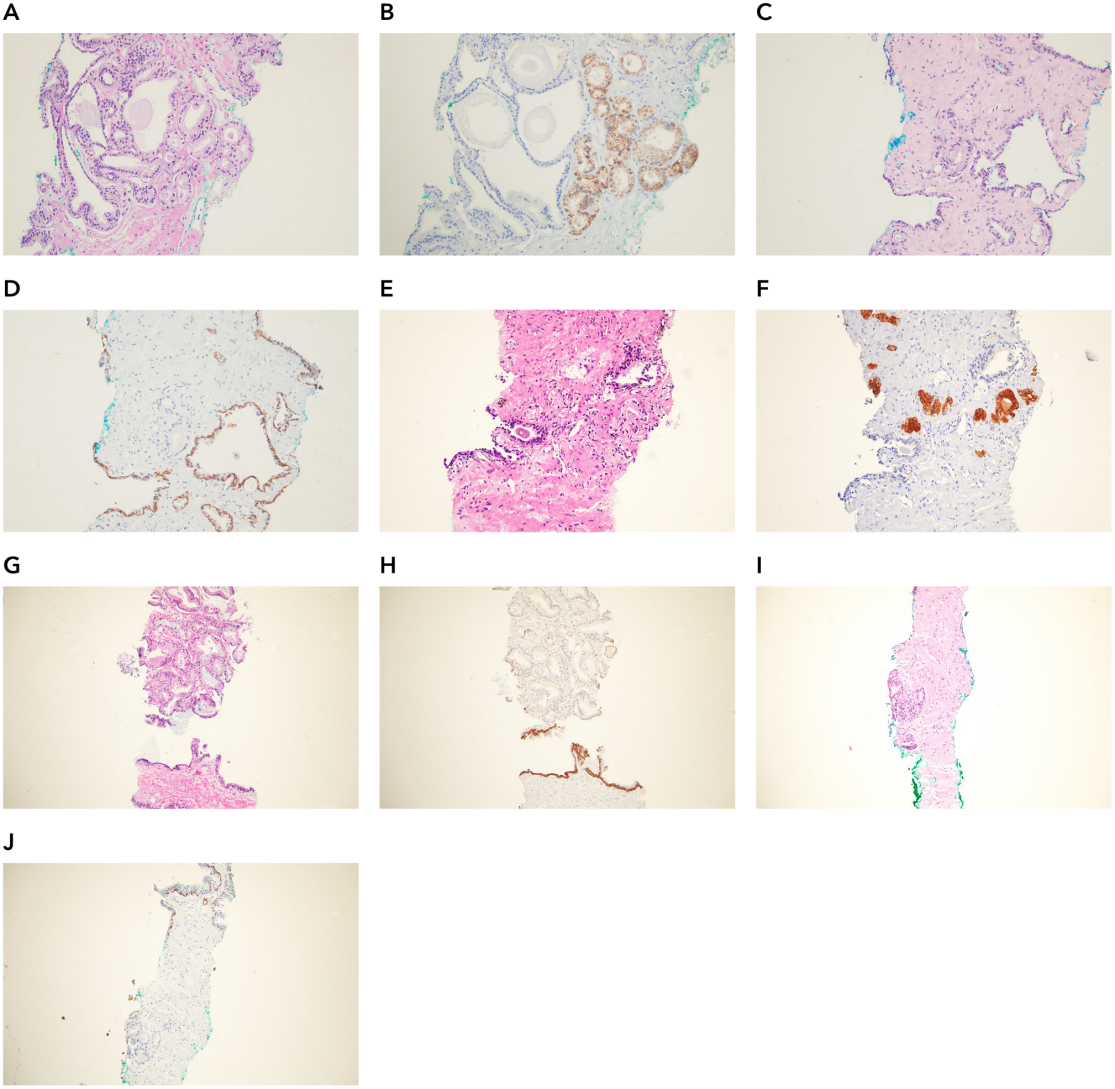
Histologic features of prostate biopsies where Paige Prostate rendered a diagnosis of malignant and the local and central pathologists rendered a diagnosis of benign or suspicious. (A, C, E, G, I) Histologic features of samples identified as suspicious by Paige Prostate but not diagnosed by the local pathologist, independent central pathologists individually, or the consensus diagnosis of the central pathologists (WSI numbers: 1002423, 1002559, 1002612, 1002603, and 1002210). (B) Expression of P504S in areas of adenocarcinoma shown in A. (D) Lack of expression of HMWC – 34βE12 in areas of adenocarcinoma shown in C. (F) Expression of P504S in areas of adenocarcinoma shown in E. (H) Lack of expression of HMWC – 34βE12 in areas of adenocarcinoma shown in G. (J) Lack of expression of HMWC – 34βE12 in areas of adenocarcinoma shown in I.

Paige Prostate rendered a diagnosis of suspicious, whereas the original consensus diagnosis of the central pathologists was ‘benign’ for 39 (39/579, 6.74%) part‐specimens. Of these, 11 (28.21%) in fact contained either cancer (Figure 5A–5J) or lesions that would require further clinical intervention (e.g. atypical small acinar proliferation). Nine of these 11 slides had lesions that were International Society of Urological Pathology Grade Group (ISUP GG) 1 and ranged in size from 0.2 to 0.4 mm, and two of these 11 slides were ISUP GG 2 and ranged in size from 0.4 to 0.7 mm. Conversely, 27 (27/39, 69.2%) were found to have a ground truth diagnosis of benign. Detailed characterization of these part‐specimens following histologic review and IHC analysis revealed that 14 were classified as atrophy, six as benign prostate tissue, one as atrophy and apical prostate tissue, three as apical prostate tissue, two as adenosis, and one as post‐atrophic hyperplasia (Figure 4A–Za). One of the discordances was found to stem from a coding error, resulting from a transcription error of the original consensus diagnosis into the database. Thus, of the original 42 discordant reads, the final discordant number of reads was 41 as depicted in Figure 1. Furthermore, as a result of the above investigations, the discordance between Paige Prostate and final ground truth was 29. At the slide level, the area under the receiver operating characteristic curve (AUC) of Paige Prostate 1.0 was 0.996 on the final ground truth.

At the patient level, Paige Prostate displayed an optimal sensitivity (1.0; CI 0.93–1.0) and NPV (1.0; CI 0.91–1.0), with only a limited number of patients being flagged as suspicious when the patient had a final ground truth benign diagnosis, resulting in a specificity of 0.78 (CI 0.64–0.89). Importantly, Paige Prostate did not fail to identify any of the patients with prostate cancer. There were, however, four significant findings at the patient level: namely, patients with only one part‐specimen containing foci of malignancy correctly identified by Paige Prostate but not diagnosed by the local or central pathologists. Of these four patients, three (Figure 5A–F from patients 7, 14, and 35) would have undergone a fundamental change in the diagnosis, as the part‐specimen diagnosed as suspicious by Paige Prostate was the sole part‐specimen containing prostate cancer or a suspicious diagnosis. Despite the change from a benign to a malignant diagnosis, it should be noted that the cancers not detected by the two central pathologists and correctly classified as suspicious by Paige Prostate were ISUP GG 1/Gleason 6 (3 + 3) prostate adenocarcinomas. In the fourth patient (Figure 5G,H from patient 42), another part‐specimen was found to harbor atypical small acinar proliferation (ASAP), which was originally detected by Paige Prostate and by the pathologists for this patient. Based on this additional finding stemming from Paige Prostate, the patient would be recommended for an additional biopsy for a confirmatory diagnosis. Paige Prostate identified correctly a focus of malignancy not diagnosed by the local or central pathologists in a fifth patient (Figure 5I,J from patient 84); however, this patient had another part‐specimen found to harbor cancer, which was originally detected by both Paige Prostate and the pathologists. Therefore, at patient level, this would not result in clinical impact. Hence, at patient level, none of the patients would have had a false‐negative diagnosis; four additional patients would have been correctly upgraded from a benign to a malignant diagnosis; and a transcription error would have been captured.

The final discordant 41 part‐specimens were re‐reviewed by an expert GU pathologist (VR). In brief, for 39 part‐specimens that Paige Prostate classified as suspicious, the expert GU pathologist rendered the diagnosis of malignant in seven; benign in 18; and deferred due to a combination of either small size of the suspicious area, need for resorting to IHC, and/or suboptimal image resolution in 14. For two part‐specimens which Paige Prostate classified as benign, the GU pathologist classified one part‐specimen as malignant and the other part‐specimen as suspicious with need of IHC. These two part‐specimens were also annotated as malignant by the central pathologists. Considering the consensus diagnosis of the central pathologists, for 28 part‐specimens where the consensus diagnosis of the central pathologists was benign, the GU pathologist rendered the diagnosis of malignant in three; benign in 16; and deferred due to a combination of either small size of the suspicious area, need of resorting to IHC, and/or suboptimal image resolution in nine. For the 13 part‐specimens where the consensus was malignant, the GU pathologist rendered the diagnosis of malignant in five WSIs; benign in two; and deferred due to a combination of either small size of the suspicious area, need of resorting to IHC, and/or suboptimal image resolution in six. It is noteworthy to mention that the central pathologists were aided by the use of IHC for the final consensus on the discordant WSIs. The expert GU pathologist classified the four patients with only one part‐specimen containing foci of malignancy correctly identified by Paige Prostate but not diagnosed by the local or central pathologists as malignant in one part‐specimen and suspicious requiring IHC for final diagnosis in the other three part‐specimens.

**Figure 6:**
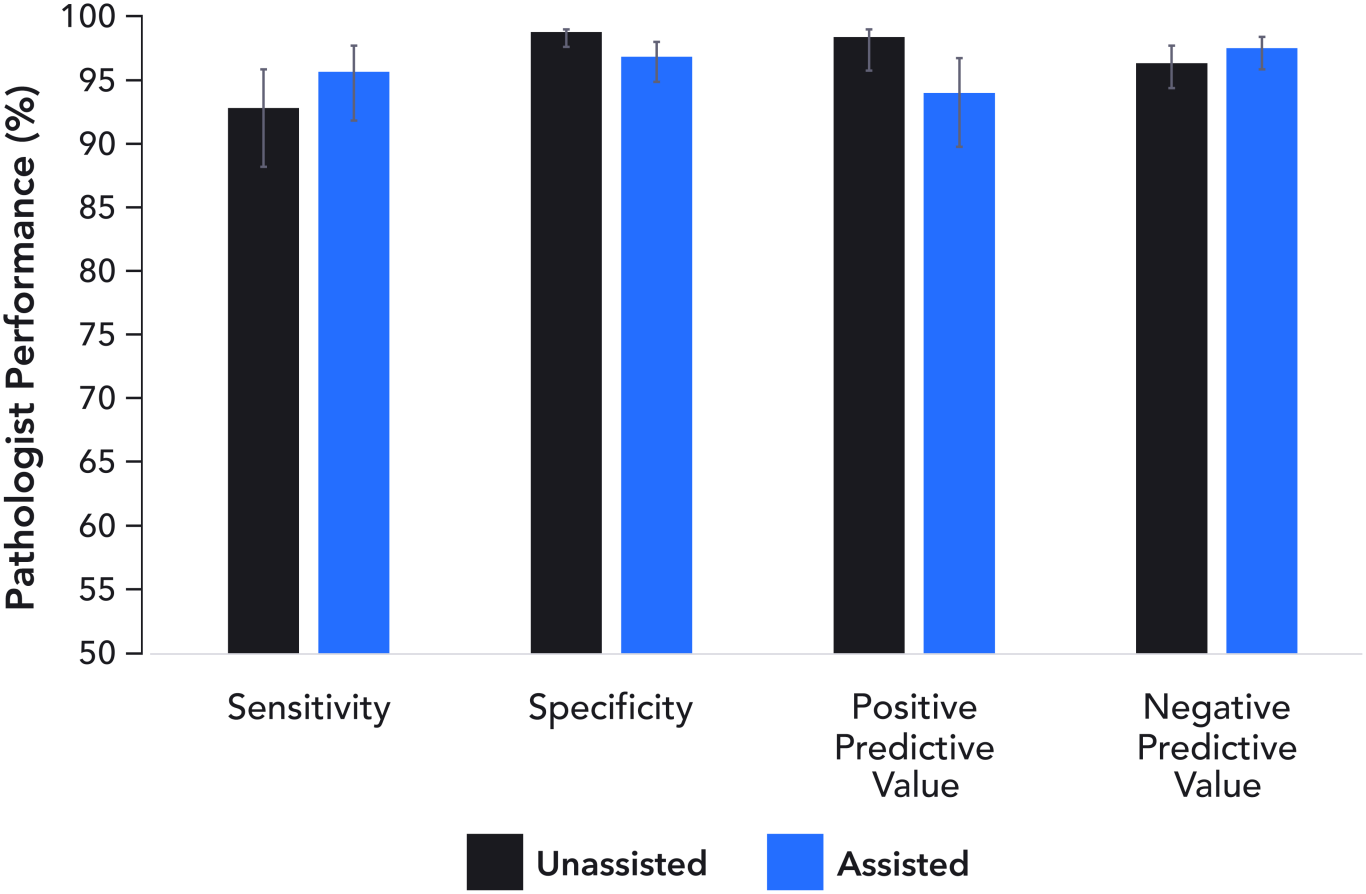
Sensitivity, specificity, positive predictive value (PPV), and negative predictive value (NPV) at the part‐specimen level of the consensus of central pathologists without and with Paige Prostate.

An exploratory assessment of the impact of Paige Prostate on the diagnostic performance of the pathologists was performed (supplementary material, Table S3). Compared with the original consensus diagnosis of two central pathologists, the consensus diagnosis based on a reanalysis in conjunction with Paige Prostate resulted in a numerical but not significant increase of 2.9% in sensitivity from 0.94 (CI 0.89–0.97) to 0.97 (CI 0.93–0.99, *p* = 0.307), and a non‐significant decrease of 2.0% in specificity from 1.0 (CI 0.99–1.0) to 0.98 (CI 0.96–0.99, *p* = 0.173; Figure 6) at a part‐specimen level. None of the patients, however, would have had an incorrect malignant diagnosis rendered; the decreased specificity stemmed from additional cases being subjected to IHC and supplementary histopathology evaluation. This was also the first time that the pathologists had used Paige Prostate.

Finally, we sought to define the potential impact of Paige Prostate on the average time spent by each of the two pathologists to review and render a diagnosis for each of the glass slides and WSI. The median time spent per glass slide and WSI was 1 min 38 s and 2 min 2 s, respectively, including completion of the report. This would result in 15.76 h per pathologist looking at all the glass slides and 19.6 h looking at all WSIs, assuming 579 WSIs to be analyzed. Given the optimal NPV of Paige Prostate at the patient level, we simulated a scenario where only the WSIs from parts classified as suspicious for cancer by Paige Prostate were histologically assessed by the pathologists (200/579 WSIs). This would result in an average diagnostic time of 6.77 h, a reduction of 65.50% of the diagnostic time for the full set of 579 WSIs.

## Discussion

Anatomic pathology is a core specialty for the multidisciplinary management of oncology patients. As such, there are enormous pressures on pathologists for not only accurate but also timely diagnoses. This pressure is compounded by the increasing number of oncology patients and the unchanged or decreased number of qualified pathologists in certain settings [1]. AI‐based approaches to address these unmet needs in the context of diagnostic prostate cancer diagnosis have showed initial promise [7, 8, 9, 10, 12, 13, 14, 15]. Here, we provide evidence supporting the notion that Paige Prostate may safely decrease pathology workload without compromising diagnostic quality.

The quality of histopathology preparations across healthcare providers varies [16], and in some cases, AI systems have failed to generalize across hospitals [7]. This study, however, was performed with materials (i.e. WSIs) obtained from the original diagnostic slides cut and stained in a laboratory not involved in the development of the AI‐based system and located in a separate country, indicating that Paige Prostate is capable of generalizing effectively across institutions. When tested against ground truth diagnoses in this real‐world dataset, Paige Prostate was found to have high sensitivity and NPV for the detection of prostate cancer in TRUS biopsies, supporting the notion that this AI‐based assay might be useful to define which TRUS biopsy slides may not require detailed histologic review, given that their probability of containing cancer would be negligible. In addition, Paige Prostate might be employed in the context of quality assurance schemes, whereby only TRUS biopsy slides with a suspicious diagnosis would be subsequently reviewed.

When assessed against the ground truth, Paige Prostate resulted in the identification of 13 instances where three experienced pathologists rendered an incorrect diagnosis (2.25% of 579 parts). The use of this AI‐based test resulted in the identification of 11 new suspicious part‐specimens (two of these with ISUP GG 2 cancers) that were initially diagnosed as benign by three pathologists with over 15 years' post‐board certification, one benign part‐specimen that was initially diagnosed as malignant by the pathologists, and one transcription error. Data on the true rate of false‐negative prostate biopsies are scant; however, it is estimated to range from 1% to 4% based on routine assessment of H&Es [3, 10, 11]. Consistent with these findings, in the current study and using the ground truth adopted combining H&E and IHC assessment, the false‐negative rate of the consensus diagnosis by two central pathologists with over 15 years' post‐board certification that was mitigated by Paige Prostate was 1.90% (11 of 579 WSIs). It should be noted, however, that original H&E assessment by Paige Prostate prompted the additional IHC analysis to define the ground truth. Given the high sensitivity and NPV of Paige Prostate (supplementary material, Table S3), and its specificity of 0.78 (without a disproportionate number of patients whose biopsies proved to be benign), Paige Prostate would result in a substantial reduction of the workload of pathologists without compromising diagnostic quality. At a 12.1% prevalence of prostate cancer [17], the use of this automated AI‐based test as an ancillary diagnostic approach (e.g. pre‐screening of the WSIs that need to be reviewed) would result in at least 60 of 100 patients being triaged without full histologic review.

This study has several limitations. First, the ground truth defined for this study was based on the use of additional ancillary tests. Albeit not infallible, this approach follows the current best practices for prostate cancer diagnosis [3, 10, 11]. Second, additional optimization of Paige Prostate 1.0 was not allowed, so we cannot rule out that higher specificity and PPV could be attained if further refinements of the system were made. Third, not all part‐specimens were analyzed for a small set of patients, due to technical issues with scanning or image transfer: one of six part‐specimens was not analyzed for 11 patients; two of six part‐specimens were not reviewed for two patients; and three of six part‐specimens were not reviewed for two patients. Given that some protocols require 18 prostate needle core biopsies per patient, our results at the patient level may constitute only a conservative estimate of the sensitivity and NPV of this AI‐based test. Fourth, the version of Paige Prostate employed in this study was designed to provide a binary classification of TRUS prostate biopsies into benign or suspicious; it was neither trained to provide more specific descriptive diagnoses (e.g. benign prostate tissue, high‐grade prostatic intra‐epithelial neoplasia, atypical small acinar proliferation) nor trained to grade the prostate cancers detected. Future studies reporting on the development and subsequent validation of Paige Prostate in these diagnostic contexts are warranted. Fifth, the reduction of diagnostic time reported in this study was inferred on the basis of average times for the histologic review of a given TRUS prostate biopsy and may have overestimated the reduction in time provided by Paige Prostate, given the time needed for slide scanning, WSI transferring, and Paige Prostate processing. We acknowledge that the deployment of Paige Prostate for screening in a pathology laboratory may increase the turnaround time for reporting of benign prostate biopsies, albeit reducing the total workload volume for the pathologists to report. This scenario will allow extra time for pathologists not only to focus on the reporting of the malignant cases but also to perform other laboratory activities. We are confident, however, that the steps necessary for Paige Prostate deployment can be optimized in a way that their impact on the diagnostic activities would be limited. Finally, the implementation of AI‐based ancillary assays, such as Paige Prostate, requires the availability of a digital pathology infrastructure and a distribution system that is cost‐effective. Given the increasing adoption of digital pathology in the diagnostic arena, future studies are warranted to define the feasibility of and the ideal paths for the implementation of Paige Prostate in both academic diagnostic pathology departments and private laboratories.

Based on an independent, real‐world cohort of 100 patients subjected to TRUS prostate biopsies, this study confirms the optimal sensitivity and NPV of Paige Prostate for the automated identification of patients whose histologic sections or WSIs would not need to be reviewed by pathologists. The deployment of Paige Prostate would have prompted a re‐review by the central pathologists of WSIs of four patients (4%), whose diagnoses would have been upgraded to a malignant category. Although these were ISUP GG 1/Gleason 6 (3 + 3) cancers with small tumor size, such patients would likely be referred to further clinical investigation with imaging studies such as imaging and/or engagement in an active surveillance program. Other aspects to take into consideration in this scenario are the legal liability of missing small cancer foci and the lack of widespread availability of expert GU pathologists for community laboratories. Given its optimal sensitivity and NPV, Paige Prostate may be considered as an ancillary test for the diagnosis of prostate cancer in TRUS core needle prostate biopsies. Whilst appropriate regulation for AI‐based systems in pathology is evolving, it is becoming evident that accreditation agencies, government bodies, and additional healthcare stakeholders need to engage in discussions with the clinical and scientific community to enable the deployment of safe and effective automated AI‐based systems for cancer screening that have the potential to improve patient care [18, 19]. Our findings may provide the basis for the future development of AI systems that could be employed in automated quality control schemes and automated screening of TRUS prostate biopsies, whereby only those flagged by the system as suspicious would require review by a diagnostic pathologist, helping to mitigate the shortage of qualified pathologists and optimize the allocation of diagnostic time and effort.

## Author contributions statement

LMS, EMP, CGF, LG, TJF and JSR‐F were responsible for study conception and design. LMS, EMP, PGOS, CK, JS, RC and RG acquired data. LMS, EMP, PGOS, CK, JS, GD and VR analyzed data. RG, RC, JK, AC, JV, BR, CK and TJF were responsible for AI algorithm design and implementation. LMS, EMP, PGOS, CGF, BF, CK, JS, LG and JSR‐F interpreted data. LMS, EMP, JS, JSR‐F and CK drafted the manuscript. All the authors reviewed manuscript and gave final approval.

## Supporting information


Supplementary materials and methods

**Table S1.** REMARK guidelines checklist

**Table S2.** Performance comparison between the local pathologists, the two independent central pathologists, the consensus of the central pathologists, and Paige Prostate

**Table S3.** Consensus of the performance of the central pathologists by assessment mode (without and with Paige Prostate)

## Data Availability

The WSIs included in this study and the consensus diagnoses of the two central pathologists are available using Microsoft Teams. Those interested in accessing the images can complete the request form https://bit.ly/36cPf6k.
