# Supplementary material for: Independent real‐world application of a clinical‐grade automated prostate cancer detection system
Source: J Pathol. 2021 Apr 27;254(2):147–58. doi: 10.1002/path.5662 (PMC8252036; doi:10.1002/path.5662)
Supplement: Supplementary file 1 — Supplementary materials and methods [file PATH-254-147-s002.docx]

**Independent real-world application of a clinical-grade automated prostate cancer detection system**

LM da Silva *et al. J Pathol* DOI: 10.1002/path.5662

**Supplementary materials and methods**

Reference numbers refer to the main text list

**Paige Prostate 1.0**

In brief, it was trained on 32 341 prostate biopsy slides from 6775 patients scanned at 20× magnification. All slides used for development were prepared at Memorial Sloan Kettering Cancer Center. As in Campanella *et al* [7], the system first preprocesses a WSI by splitting it up into a collection of smaller images, which are known as tiles. For Paige Prostate 1.0, each WSI is split into 224 × 224 RGB tiles. For each tile, a simple test for determining if it is background is then done, which consists of identifying tiles in which the pixel intensity variance within the 224 × 224 tiles is below a threshold. All background tiles are ignored from subsequent processing during inference or training. On average, each WSI has 4693 non-background tiles. The training happens in two stages: learning the backbone Convolutional Neural Network (CNN) and learning a CNN for aggregating tile-level features. The backbone CNN was an SE-ResNet50 architecture and was trained for 20 epochs using multiple instance learning (MIL) with an initial learning rate of 0.0002 with a learning rate decay of 0.5 every 10 epochs. Tiles were augmented using standard techniques such as random rotations, sharpening, and Hue Saturation Value (HSV) shift. During the forward pass, MIL computes the current estimated probability of suspicion for cancer on all non-background tiles for each slide in a specimen. The five tiles with the greatest probability are then selected for updating during the backward pass. Ideally, this means that for positive slides, it will choose tiles with tumors, but for negative slides it will choose tiles that look the most like tumors but are not. After training, the SE-ResNet50 CNN is used to extract 2048 dimensional embeddings from each tile. These embeddings were fed into another CNN that has two layers of 1 × 1 convolutions followed by the rectified linear activation function (ReLU). This is followed by global soft attention-based pooling to aggregate information across each specimen, followed by an output layer with a logistic sigmoid activation function. This aggregator CNN was trained for 96 epochs with a learning rate of 0.0001. The key differences between Paige Prostate 1.0 and the earlier versions are the use of SE-ResNet50 and using the aggregator neural network described above, whereas Paige Prostate Alpha used ResNet34 and a simple recurrent neural network for slide-level prediction. After development was completed using the slides from Memorial Sloan Kettering, Paige Prostate 1.0 was locked, and no further optimization was done on additional slides.

**Details of the 20 patients subjected to additional IHC analysis**

With the aim to ensure accuracy of the principles employed to define the ground truth diagnoses, 113 randomly selected slides related to 20 patients where the diagnoses were concordant between the local pathologist, central pathologists, and Paige Prostate were retrieved. The slide pool of the 20 patients was comprised of one patient with 9 slides, one patient with 8 slides, four patients with 7 slides, five patients with 6 slides, four patients with 5 slides, three patients with 4 slides, and two patients with 3 slides, totaling 113 slides. Some of the patients had fewer than six concordant slides analyzed because the remaining set of their non-analyzed slides was in the pool of the discordant slides. One patient had one slide not sent for this study from the archive of the pathology department.
