# Supplementary material for: Independent real‐world application of a clinical‐grade automated prostate cancer detection system
Source: J Pathol. 2021 Apr 27;254(2):147–58. doi: 10.1002/path.5662 (PMC8252036; doi:10.1002/path.5662)
Supplement: Supplementary file 2 — Table S1. REMARK guidelines checklist [file PATH-254-147-s003.docx]

**Independent real-world application of a clinical-grade automated prostate cancer detection system**

LM da Silva *et al. J Pathol* DOI: 10.1002/path.5662

**Table S1.** REMARK guidelines checklist

| **Section & Topic** | **No** | **Item** | **Reported on manuscript page number:** |
| --- | --- | --- | --- |
|  |  |  |  |
| **TITLE** | **1** | **Independent real-world application of a clinical-grade automated prostate cancer detection system** | 1 |
| **ABSTRACT** | **2** | Structured summary of background, methods, results, and conclusions | 2 |
| **INTRODUCTION** |  |  |  |
|  | **3** | Scientific and clinical background:   - Artificial intelligence (AI)-based systems applied to whole histopathology slide images (WSIs) have the potential to mitigate challenges posed by diagnostic variability, histopathology caseload, and shortage of pathologists - ‘Paige Prostate’ is a clinical-grade weakly supervised deep learning AI test that has recently received Breakthrough Designation for the automated detection of cancer in prostate biopsies | 3 |
|  | **4** | Study objectives**:**   - to assess the diagnostic performance of this AI system in WSIs of TRUS prostate biopsies, to define its impact on the accuracy of board-certified pathologists interpreting these WSIs - to evaluate its impact on the diagnostic accuracy and efficiency of experienced diagnostic pathologists | 3 |
| **METHODS** |  |  |  |
| *Study design* | **5** | Assessments of sensitivity, specificity, positive (PPV) and negative (NPV) predictive values of a local pathologist, two central pathologists, and Paige Prostate in the diagnosis of 600 transrectal ultrasound-guided prostate needle core biopsy regions (‘part-specimens’) from 100 consecutive patients, and to ascertain the impact of Paige Prostate on diagnostic accuracy and efficiency of experienced diagnostic pathologists | 4, 5, 6, and 7  Figure 1 |
| *Participants* | **6** | Eligibility criteria:  - Archival hematoxylin-and-eosin (H&E)-stained histologic sections with good staining quality standards for optical microscopy reading | 4 |
|  | **7** | Where and when potentially eligible participants were identified (setting, location, and dates)  - Instituto Mario Penna in Brazil (Belo Horizonte city)  - Between May 9, 2019 and August 22, 2019 | 4 |
|  | **8** | Participants formed a consecutive series | 4 |
| *Test methods* | **9** | Reference standard: central pathologists consensus diagnosis | 7 |
|  | **10** | Rationale for choosing the reference standard (if alternatives exist): current standard of medical practice for most Cancer Centers | 7 |
|  | **11** | Definition of ground truth | 7 |
|  | **12** | No clinical information and previous results were available  to the performers/readers of Paige Prostate | 4 |
|  | **13** | No clinical information and previous results were available  to the central pathologist before reporting | 4 |
| *Analysis* | **14** | The following procedure was used to assign the ground truth labels to each part. If the consensus of the central pathologists and Paige Prostate agreed, then this classification was assigned as the ground truth for the part-specimen; otherwise, additional histologic sections of the corresponding part were cut and subjected to IHC analysis and reinterpretation by the pathologists to assign the final ground truth. For WSIs with compromised viewing quality, the glass slides were reviewed | 6,7 |
|  | **15** | The analyses were completed (i) by treating each part (i.e. specific region targeted by a TRUS prostate needle core biopsy) as independent (i.e. part-specimen level) and (ii) by treating the diagnosis for a patient based on all parts for a given patient (i.e. patient level). *P* values ≤ 0.05 on a two-sided exact binomial test were considered significant and two-sided 95% confidence intervals (CIs) were calculated  In addition, one hundred (100) concordant part-specimens (classification of Paige and that of the consensus diagnosis of the central pathologists were concordant) were re-reviewed by an independent general pathologist (PR). In addition, discordant part-specimens between Paige Prostate or the consensus of the central pathologists and the final ground truth were digitally re-reviewed by an expert GU pathologist | 6, 7 |
|  | **16** | How missing data were handled | Figure 1 |
| **RESULTS** |  |  |  |
| *Participants* | **17** | Flow of participants, using a diagram | Figure 1 |
|  | **18** | Baseline demographic and clinical and pathological characteristics of participants | Table 1 |
|  | **19** | Distribution of severity of disease in those with the target condition | N/A* |
|  | **20** | Distribution of alternative diagnoses in those without the target condition | N/A* |
|  | **21** | Time interval and any clinical interventions between index test and reference standard | N/A* |
| *Test results* | **22** | Cross tabulation of the results (or their distribution)  by the results of the reference standard  Estimates of diagnostic accuracy and their precision (such as 95% confidence intervals) | Figures 2 and 6  Supplemental tables 1 and 2 |
| **DISCUSSION** |  |  |  |
|  | **23** | Study limitations:   - The ground truth defined for this study was based on the use of additional ancillary tests; however, these were not performed in all cases where agreement between the independent pathologists and Paige Prostate was observed. This approach follows the current best practices for prostate cancer diagnosis. Instead, 113 randomly selected slides related to 20 patients where the diagnoses were concordant between the local pathologist, central pathologists, and Paige Prostate were subjected to IHC analysis to ensure the accuracy of the ground truth diagnoses - Additional optimization of Paige Prostate was not allowed, so we cannot rule out that higher specificity and PPV could be attained if further refinements of the system were performed. Third, - Not all part-specimens were analyzed for a small set of patients, due to technical issues with scanning or image transfer - The reduction of diagnostic time reported in this study was inferred on the basis of average times for the histologic review of a given TRUS prostate biopsy, and may have overestimated the reduction in time provided by Paige Prostate, given the time needed for slide scanning, WSI transferring, and Paige Prostate processing. We acknowledge that the deployment of Paige Prostate for screening in a pathology laboratory may increase the turnaround time for reporting of benign prostate biopsies, albeit reducing the total workload volume for the pathologists to report. This scenario will allow extra time for pathologists not only to focus on the reporting of the malignant cases but also to perform other laboratory activities. We are confident, however, that the steps necessary for Paige Prostate deployment can be optimized in a way that their impact on the diagnostic activities would be limited | 13 and 14 |
|  | **24** | Implications for practice, including the intended use:   - The deployment of Paige Prostate would have prompted a re-review by the central pathologists of WSIs of four patients (4%), whose diagnoses would have been upgraded to a malignant category, albeit of all them being ISUP 1 / Gleason 6 (3+3) cancers - Given its optimal sensitivity and NPV, Paige Prostate may be considered as an ancillary test for the diagnosis of prostate cancer in TRUS core needle prostate biopsies - Our findings may provide the basis for the future development of AI systems that could be employed in the automated quality control schemes and automated screening of TRUS biopsies, whereby only those flagged by the system as suspicious would require review by a diagnostic pathologist, helping to mitigate the shortage of qualified pathologists and optimize the allocation of diagnostic time and effort | 15 and 16 |
| **OTHER INFORMATION** |  |  |  |
|  | **25** | Registration number and name of registry | N/A* |
|  | **26** | Where the full study protocol can be accessed | N/A* |
|  | **27** | Sources of funding and other support; role of funders | 16 |
|  |  |  |  |

*N/A, not applicable.
