# Supplementary material for: Independent real‐world application of a clinical‐grade automated prostate cancer detection system
Source: J Pathol. 2021 Apr 27;254(2):147–58. doi: 10.1002/path.5662 (PMC8252036; doi:10.1002/path.5662)
Supplement: Supplementary file 3 — Table S2. Performance comparison between the local pathologists, the two independent central pathologists, the consensus of the central pathologists, and Paige Prostate [file PATH-254-147-s004.docx]

**Independent real-world application of a clinical-grade automated prostate cancer detection system**

LM da Silva *et al. J Pathol* DOI: 10.1002/path.5662

**Table S2.** Performance comparison between the local pathologists, the two independent central pathologists, the consensus of the central pathologists, and Paige Prostate

| **Assessment** | **Part-specimen level (*N* = 579)** | | | | **Patient level (*N* = 100)** | | | |
| --- | --- | --- | --- | --- | --- | --- | --- | --- |
|  | **Sensitivity** | **Specificity** | **PPV** | **NPV** | **Sensitivity** | **Specificity** | **PPV** | **NPV** |
| Local  pathologist (manual) | 87.4% (153/175)  (81.6%, 92.0%)  *p =* 0.012 | 98.5% (398/404)  (96.8%, 99.5%)  *p =* 0.022 | 96.2% (153/159)  (92.0%, 98.6%)  *p =* 0.030 | 94.8% (398/420)  (92.2%, 96.7%)  *p =* 0.014 | 88.0% (44/50)  (75.7%, 95.5%)  *p =* 0.205 | 100.0% (50/50)  (92.9%, 100.0%)  *p =* 0.035 | 100.0% (44/44)  (92.0%, 100.0%)  *p =* 0.037 | 89.3% (50/56)  (78.1%, 96.0%)  *p =* 0.212 |
| Independent central  pathologist  1 (manual) | 92.0% (161/175)  (86.9%, 95.6%)  *p =* 0.107 | 97.8% (395/404)  (95.8%, 99.0%)  *p =* 0.078 | 94.7% (161/170)  (90.2%, 97.6%)  *p =* 0.078 | 96.6% (395/409)  (94.3%, 98.1%)  *p =* 0.116 | 94.0% (47/50)  (83.5%, 98.7%)  *p =* 0.616 | 94.0% (47/50)  (83.5%, 98.7%)  *p =* 0.254 | 94.0% (47/50)  (83.5%, 98.7%)  *p =* 0.272 | 94.0% (47/50)  (83.5%, 98.7%)  *p =* 0.632 |
| Independent central  pathologist  2 (manual) | 89.7% (157/175)  (84.2%, 93.8%)  *p =* 0.032 | 99.5% (402/404)  (98.2%, 99.9%)  *p* < 0.001 | 98.7% (157/159)  (95.5%, 99.8%)  *p* < 0.001 | 95.7% (402/420)  (93.3%, 97.4%)  *p =* 0.043 | 90.0% (45/50)  (78.2%, 96.7%)  *p =* 0.285 | 100.0% (50/50)  (92.9%, 100.0%)  *p =* 0.034 | 100.0% (45/45)  (92.1%, 100.0%)  *p =* 0.035 | 90.9% (50/55)  (80.0%, 97.0%)  *p =* 0.306 |
| Independent  central pathologist  1 (digital) | 95.4% (167/175)  (91.2%, 98.0%)  *p =* 0.430 | 96.5% (390/404)  (94.3%, 98.1%)  *p =* 0.155 | 92.3% (167/181)  (87.4%, 95.7%)  *p =* 0.145 | 98.0% (390/398)  (96.1%, 99.1%)  *p =* 0.428 | 94.0% (47/50)  (83.5%, 98.7%)  *p =* 0.626 | 84.0% (42/50)  (70.9%, 92.8%)  *p =* 0.736 | 85.5% (47/55)  (73.3%, 93.5%)  *p =* 0.737 | 93.3% (42/45)  (81.7%, 98.6%)  *p =* 0.640 |
| Independent  central pathologist  2 (digital) | 93.7% (164/175)  (89.0%, 96.8%)  *p* = 0.191 | 99.8% (403/404)  (98.6%, 100.0%)  *p* < 0.001 | 99.4% (164/165)  (96.7%, 100.0%)  *p* < 0.001 | 97.3% (403/414)  (95.3%, 98.7%)  *p* = 0.201 | 94.0% (47/50)  (83.5%, 98.7%)  *p =* 0.607 | 98.0% (49/50)  (89.4%, 99.9%)  0.050 | 97.9% (47/48)  (88.9%, 99.9%)  *p =* 0.051 | 94.2% (49/52)  (84.1%, 98.8%)  *p =* 0.609 |
| Consensus of central pathologists (digital) | 93.7% (164/175)  (89.0%, 96.8%)  *p =* 0.193 | 99.8% (403/404)  (98.6%, 100.0%)  *p* < 0.001 | 99.4% (164/165)  (96.7%, 100.0%)  *p* < 0.001 | 97.3% (403/414)  (95.3%, 98.7%)  *p =* 0.198 | 94.0% (47/50)  (83.5%, 98.7%)  *p =* 0.606 | 98.0% (49/50)  (89.4%, 99.9%)  *p =* 0.051 | 97.9% (47/48)  (88.9%, 99.9%)  *p =* 0.059 | 94.2% (49/52)  (84.1%, 98.8%)  *p =* 0.610 |
| Paige Prostate | 98.9% (173/175) (95.9%, 99.9%) | 93.3% (377/404) (90.4%, 95.5%) | 86.5% (173/200) (81.0%, 90.9%) | 99.5% (377/379) (98.1%, 99.9%) | 100.0% (50/50) (92.9%, 100.0%) | 78.0% (39/50) (64.0%, 88.5%) | 82.0% (50/61) (70.0%, 90.6%) | 100.0% (39/39) (91.0%, 100.0%) |

*P* value is based on bootstrap analysis of paired differences compared with Paige Prostate. () Two-sided exact binomial 95% CI.
