# Supplementary material for: Independent real‐world application of a clinical‐grade automated prostate cancer detection system
Source: J Pathol. 2021 Apr 27;254(2):147–58. doi: 10.1002/path.5662 (PMC8252036; doi:10.1002/path.5662)
Supplement: Supplementary file 4 — Table S3. Consensus of the performance of the central pathologists by assessment mode (without and with Paige Prostate) [file PATH-254-147-s001.docx]

**Independent real-world application of a clinical-grade automated prostate cancer detection system**

LM da Silva *et al. J Pathol* DOI: 10.1002/path.5662

**Table S3.** Consensus of the performance of the central pathologists by assessment mode (without and with Paige Prostate)

|  |  | **Part-specimen level (*N* = 579)** | | | **Patient level (*N* = 100)** | | |
| --- | --- | --- | --- | --- | --- | --- | --- |
| **Measure** | **Assessment** | **Estimate (*n*/*N*), 95% CI*** | | **Difference,**  **95% CI^†^**  **(*P* value)^†^** | **Estimate (*n*/*N*), 95% CI*** | | **Difference,**  **95% CI^†^**  **(*P* value)^†^** |
|  |  | **Unassisted** | **PAIGE assisted** |  | **Unassisted** | **PAIGE assisted** |  |
| Sensitivity | Consensus Read | 93.7%  (89.0%, 96.8%) | 96.6%  (92.7%, 98.7%) | 2.9%  (0.0%, 8.5%)  *p =* 0.307 | 94.0%  (83.5%, 98.7%) | 96.0%  (86.3%, 99.5%) | 2.0%  (0.0%, 12.5%)  *p =* 1.000 |
| Specificity | Consensus Read | 99.8%  (98.6%, 100.0%) | 97.8%  (95.8%, 99.0%) | −2.0%  (−4.9%, 0.0%)  *p =* 0.173 | 98.0%  (89.4%, 99.9%) | 92.0%  (80.8%, 97.8%) | −6.0%  (−23.1%, 7.7%)  *p =* 0.739 |
| PPV | Consensus Read | 99.4%  (96.7%, 100.0%) | 94.9%  (90.6%, 97.7%) | -4.5%  (−10.9%, 0.1%)  *p =* 0.189 | 97.9%  (88.9%, 99.9%) | 92.3%  (81.5%, 97.9%) | −5.6%  (−21.4%, 7.1%)  *p =* 0.751 |
| NPV | Consensus Read | 97.3%%  (95.3%, 98.7%) | 98.5%  (96.8%, 99.4%) | 1.2%  (−0.1%, 3.4%)  *p =* 0.317 | 94.2%  (84.1%, 98.8%) | 95.8%  (85.7%, 99.5%) | 1.6%  (−1.8%, 10.5%)  *p =* 1.000 |

*For individual rates, two-sided 95% exact binomial confidence interval.
^†^For difference, two-sided 95% CI and *P* value for one-sided test based on bootstrap analysis of paired assessments.
